# PHEX^L222P^ Mutation Increases *Phex* Expression in a New ENU Mouse Model for XLH Disease

**DOI:** 10.3390/genes13081356

**Published:** 2022-07-28

**Authors:** Carole El Hakam, Alexis Parenté, Fabienne Baraige, Laetitia Magnol, Lionel Forestier, Florent Di Meo, Véronique Blanquet

**Affiliations:** 1 INSERM U1094, INRAE USC1501, IRD U270, EpiMaCT-Epidemiology of Chronic Diseases in Tropical Zone, Univ. Limoges, 2 Rue Pr Descottes, F-87000 Limoges, France; carole.alhakam@hotmail.com (C.E.H.); alexis.parente@unilim.fr (A.P.); fabienne.baraige@unilim.fr (F.B.); laetitia.magnol@unilim.fr (L.M.); lionel.forestier@unilim.fr (L.F.); 2 INSERM U1248 Pharmacology & Transplantation, Univ. Limoges, 2 Rue Pr Descottes, F-87000 Limoges, France; florent.di-meo@inserm.fr

**Keywords:** phex, ENU, XLH, hypophosphatemia, bone, skeletal development

## Abstract

*Phex^L222P^* mouse is a new ENU mouse model for XLH disease due to Leu to Pro amino acid modification at position 222. *Phex^L222P^* mouse is characterized by growth retardation, hypophosphatemia, hypocalcemia, reduced body bone length, and increased epiphyseal growth plate thickness and femur diameter despite the increase in PHEX^L222P^ expression. Actually, *Phex^L222P^* mice show an increase in *Fgf23*, *Dmp1*, and *Mepe* and *Slc34a1 (Na-Pi IIa cotransporter)* mRNA expression similar to those observed in *Hyp* mice. Femoral osteocalcin and sclerostin and *Slc34a1* do not show any significant variation in *Phex^L222P^* mice. Molecular dynamics simulations support the experimental data. P222 might locally break the E217-Q224 *β*-sheet, which in turn might disrupt inter-*β*-sheet interactions. We can thus expect local protein misfolding, which might be responsible for the experimentally observed PHEX^L222P^ loss of function. This model could be a valuable addition to the existing XLH model for further comprehension of the disease occurrence and testing of new therapies.

## 1. Introduction

Hypophosphatemic rickets (HR) is a heterogeneous group of rare diseases characterized by a defect in renal handling of phosphorus, which leads to bone mineralization disorder and hypophosphatemia [1]. Several forms of HR are found and can be distinguished by their pattern of inheritance and genetic cause. The X-linked dominant form (XLH; MIM 307800) represents the most common inherited HR in humans, affecting about 1 in 20,000 people worldwide, and accounts for approximately 80% of the familial cases of hypophosphatemic rickets [2,3]. It is caused by a mutation in the phosphate-regulating gene with homologies to endopeptidases on the X-chromosome (*Phex*). A rare X-linked recessive form (XLRH; M IM300554) has been reported with a mutation in the Chloride Channel *5* gene (*CLCN5*) [4]. In a few families, hereditary hypophosphatemic rickets has had an autosomal dominant inheritance pattern (ADHR; MIM 193100) due to a mutation in the gene encoding the Fibroblast Growth Factor *23* gene (*FGF23*) [5]. HR could also be inherited in autosomal recessive form, namely ARHR1 (MIM 241520), caused by a mutation in the Dentin Matrix acidic Phosphoprotein 1 gene (*DMP1*) [6]; and ARHR2 (MIM 613312), caused by a mutation in the Ecto-Nucleotide Pyrophosphatase/Phosphodiesterase 1 gene (*ENPP1*) [7].

Another rare type of the disorder is known as hereditary hypophosphatemic rickets with hypercalciuria (HHRH). In addition to hypophosphatemia, this condition is characterized by the excretion of high levels of calcium in the urine [8].

XLH is characterized by rickets, hypophosphatemia, renal phosphate wasting, and growth retardation [9,10], but some may have normal growth, normal or aberrant vitamin D metabolism, poor mineralization of the teeth [11,12,13,14,15], hearing impairment, and muscle weakness induced by PHEX loss of function [16,17,18,19,20]. The *Phex* gene, formerly referred to as the *Pex* gene, is located on chromosome Xp22.1–22.2 in humans and on the X chromosome at 72.38 cM in mice [16,21]. The coding region spanning 2250 bp (22 exons) encodes for a type II transmembrane endopeptidase of 749 amino acids. It is organized into three domains: a small amino-terminal intracellular tail (residues 1 to 15), a short transmembrane domain (residues 16 to 58), and a large carboxyterminal extracellular domain (residues 59 to 749) that contains 10 highly conserved cysteine residues and a zinc-binding motif required for its conformation and activity. This composition shows that PHEX has significant homologies with the members of the M13 zinc-metallopeptidases family, which are involved in the degradation or activation of a variety of peptide hormones. The murine *Phex* gene shows 91% of identity at the DNA level and 96% of identity at the protein level to the human gene product [22,23,24].

*Phex* is primarily expressed in bone lineage, such as osteoblasts, osteocytes and odontoblasts, and its expression decreases with age [25,26]. PHEX was initially thought to directly inactivate FGF23, a suspected phosphaturic factor [27,28], to regulate bone and dentin mineralization and renal phosphate reabsorption. Later studies have shown that FGF23 is not a substrate of PHEX-encoded endopeptidase [29,30,31], even if the decrease in or absence of PHEX expression induces the increase in FGF23 expression [32,33]. Indeed, ASARM peptides of the SIBLINGs mineral-regulating proteins, which include proteins such as DMP-1 (Dentin Matrix Protein-1), BSP (Bone Sialoprotein), OPN (Osteopontin), DSPP (Dentin Sialophosphoprotein) and MEPE (Matrix Extracellular Phosphoglycoprotein), all of which bind strongly to hydroxyapatite mineral in the extracellular matrix of bone, are the only known physiological substrates for PHEX [34,35,36,37,38,39,40]. Released ASARM peptides play roles in mineralization, bone turnover, mechanotransduction, phosphate regulation and energy metabolism. To date, 720 mutations of *Phex* have been registered (HGMD 2020.4), from which 435 different *Phex* mutations with a phenotype annotation have been described in patients with HR, which include 164 nonsense/missense mutations, 79 splicing mutations, 46 sequence alterations, 50 small insertions, 90 small deletions, 6 indels, and in different positions along the gene (Ensembl GRCh37 release 99-January 2020 © EMBL-EBI). In parallel, 18 alleles are found in mice: 4 spontaneous (*Phex^Hyp^^-^^2J^, Phex^Hyp-Duk^* and *Phex^Hyp^, Phex^Kbus/Idr^*); 1 radiation (*Phex^Gy^*); 7 targeted (*Phex^tm1(EGFP/cre/ERT2)Wtsi^, Phex^tm1(KOMP)Vlcg^, Phex^tm1.1Mkd^*, *Phex^tm1.2Mkd^, Phex^tm1e(KOMP)Wtsi^*, Tg(ACTB-Phex)#Blan, Tg(Bglap2-Phex)1Ldq); and 6 ENU alleles (*Phex^Hpr^, Phex^m1Jrt^, Phex^Mhdabap012^, Phex^Mhdabap024^, Phex^Pug^* and *Phex^Ska1^*) (http://www.informatics.jax.org) [41]. Although they share common symptoms of the disease, the different mouse models show several manifestations and gravity levels of XLH disease [33,42,43,44,45,46]. It could be due to the carried allele [18] or the genetic background [42,45,47]. However, no single mutation is predominant in XLH disease [48]. Altogether, the mutations described in XLH patients lead to a loss of PHEX function.

Here, we report a new ENU-induced mouse model for XLH disease presenting a *Phex^L222P^* mutation. Hemizygous male and heterozygous female *Phex^L222P^* mice exhibit the classic clinical manifestations of XLH, including growth retardation, hypophosphatemia, and skeletal abnormalities. This substitution causes an overexpression of *Phex* transcript and protein, but surprisingly, it leads to a loss-of-function phenotype. We also observed a deregulation of several matrix protein expressions, as in the case of PHEX loss of function, making *Phex^L222P^* mice a valuable new model for the study of PHEX interactions in XLH disease.

## 2. Materials and Methods

**Mice**

*Phex^L222P^* mice (mixed B6;FVB background) were derived from a sensitized dominant ENU mutagenesis program performed in our lab as described previously [49]. Briefly, ENU males C57BL/6J mice were bred with *Gdf8* knockout females (generated on FVB genetic background). Once the mutant mouse line had been established, it was first maintained by backcrosses to Gdf8 knockout females, then to wildtype (WT) FVB mice to study the mutation manifestation alone, generating *Phex^L222P^*; *Gdf8*^+/+^ animals. *Phex* mutant mice were established by crossing for more than 15 generations. All tests were performed on hemizygous (*Phex^L222P^*/Y) males, heterozygous (*Phex^L222P^*/*Phex*) females, and their age-matched littermate controls. All the mice were bred and housed in the animal facility of Limoges University under controlled conditions (21 °C, 12/12 h light–dark cycle) with free access to standard mouse chow (RM1 (P) 801151, Special diets services) and tap water [50]. All animal experiments were performed according to European directives (86/609/CEE and 2010/63/UE) and approved by the Committee on Ethics of Animal Experiments from the Author’s Institution, “Comité Régional d’Ethique de l’Expérimentation Animale” of the Limousin region (n ° 10-2014-10). According to the European Directive 210-63-EU, mice were observed daily for the general health status and mortality scoring.

**Plasma biochemistry**

Blood samples (250 µL) were obtained from 8-week-old nonfasted anesthetized mice by the puncture of the retro-orbital sinus. Plasma analyses of ALP (Alkaline Phosphatases), Ca (Calcium), and Pi (inorganic Phosphorus) were executed using Konelab 30 Clinical Chemistry Analyzer with the adapted test kits (Thermo Scientific, Waltham, MA, USA) on the day of collection.

**Histological analyses**

Left femurs were quickly dissected free of most adherent tissue from 3 months old mice then fixed, decalcified, and cryoprotected as described before by Jiang et al. [51]. Cryosections of 10 µm thickness were stained with the HES staining procedure or Masson Trichrome (Light Green). The slides were scanned using Hamamatsu NanoZoomer RS 2.0 (Hamamatsu), and images were visualized using NDP view (v1.2.52).

**Whole-mount skeletal preparations**

Mice were sacrificed by CO_2_ asphyxiation and skin and internal organs were removed if possible. Skeletons were fixed in ethanol 95% then in acetic acid 100%. Skeletal morphologies were stained by Alizarin Red (A-R) and Alcian Blue (A-B) staining, followed by tissue de-staining with 20% glycerol-1% potassium hydroxide (KOH) and clarification with a glycerol series (50%, 70%) to finally preserve them in glycerol 100% for ulterior skeletal morphology analyses. To avoid the fat de-staining problem, skeletons were kept in acetone for 2–3 days before starting the de-staining. Each step lasts for 4 to 7 days under agitation at room temperature. The staining solution is a mixture of 5% A-B (0.3% Alcian Blue in ethanol 70%, H_2_O), 5% A-R (0.1% Alizarin Red in ethanol 95%), 5% Acetic Acid (100%) and 85% ethanol (70%). To measure the bone length, isolated bones were laid next to a ruler and photographed using a digital camera.

**DNA, RNA and Protein extractions**

DNA was extracted from mutant mice blood samples (200 µL) according to the manufacturer’s instructions (QIAamp DNA Blood Mini Kit, Qiagen), quantified by Nanodrop, and stored at −20 °C. Total RNA and proteins were extracted from right femur and kidney of a minimum of 3 mice from each category at 3 months of age. Samples were frozen in liquid nitrogen and stored at –80 °C for later extraction, or they were directly homogenized into fine powder in liquid nitrogen using a mortar and pestle.

RNA was extracted using TRI-reagent^®^ (Sigma-Aldrich, St. Louis, MO, USA) and treated with DNase I (Sigma-Aldrich, St. Louis, MO, USA) following manufacturer’s instructions. The quality and quantity of RNA were evaluated by the Agilent 2100 bioanalyzer and conserved at −80 °C until use.

Proteins were extracted by a protein extraction buffer: 50 mM Tris-HCl pH7.4, 5 mM EDTA pH8, 0.5% Sodium Desoxycolate, 0.5% Triton and Complete Protease Inhibitor Cocktail Tablet (Roche Diagnostics), under agitation at 4 °C; collected by centrifugation (12,000× *g*, 4 °C, 20 min), then quantified at absorbance A595 using the Bradford assay (Bio-Rad).

**Gene mapping**

A panel of 56 MITs polymorphic markers between FVB and C57BL/6J was used for a genome-wide linkage analysis as previously described by Magnol et al. (2011). PCR cycles were performed as following: 94 °C for 5 min, 35 cycles of 94 °C for 30 s, 55 °C for 45 s, and 72 °C for 45 s, and a final extension of 72 °C for 5 min. A further fine mapping was performed by HRM technology (High-Resolution Melting; Applied Biosystems, Foster City, CA, USA) according to the manufacturer’s instructions using 4 SNPs, then we identified *Phex* as a candidate gene in the reduced linkage region leading to the mutant phenotype using JAX informatics (http://www.informatics.jax.org/marker, accessed on 1 January 2021) (Mouse Genome Informatics).

**Mutation analysis**

First-strand cDNA was synthesized from femur total RNA using the high-capacity cDNA reverse transcription kit (Applied Biosystems, Foster City, CA, USA). cDNA of *Phex* gene was sequenced with overlaps primers. The mutation was confirmed by direct sequencing of the region of interest from amplified genomic DNA of many mutant mice which are then genotyped by RFLP (Restriction Fragment Length Polymorphism) assays. All primer sequences are available upon request. Mice are later classified as WT or mutant based on their tail length.

**RFLP-PCR assays**

DNA was amplified using UptiTherm DNA Polymerase (Uptima INTERCHIM) across intron 5 and exon 6 to encompass the mutation site using the forward primer 5′-GCTGGTCTGCCAATGAGTCCG-3′ and the reverse primer 5′-CACAAGGTCTCTGCTTCCTGCAC-3′. PCR cycles were performed as follows: 94 °C for 5 min, 35 cycles of 94 °C for 30 s, 58 °C for 30 s, and 72 °C for 40 s, and a final extension of 72 °C for 5 min. PCR products were digested separately with MspI (digest the mutant fragment) and AluI (digest the wildtype fragment) restriction enzymes (Biolabs) for greater certainty. Digested fragments were separated by agarose gel 3% electrophoresis before image acquisition using a Gel DocTM UV transilluminator (Bio-Rad). Digestions with MspI and AluI were made overnight using 10 µL unpurified PCR products, 2 µL enzyme buffer and 1 µL restriction enzyme.

**Real Time PCR**

Quantitative real-time RT-PCR assays were performed in triplicate for each sample using 20 ng cDNAs prepared as described above and Taqman probes (Applied Biosystems, Foster City, CA, USA) to study the expression of *Phex* (Taqman prob Mm00448119_m1) in femur and kidney and other genes that could vary according to it in femur: *Fgf23* (Taqman prob Mm00445621_m1); *Mepe* (Matrix extracellular phosphoglycoprotein, Taqman probMm02525159_s1); *Dmp1* (Taqman prob Mm01208363_m1); *Sost* (Sclerostin, Taqman prob Mm04208528_m1); *Bglap3* (Bone γ-carboxyglutamate protein 3 from the superfamily of the osteocalcin, Taqman prob Mm00649782_gH); and in kidney: *Slc34a1* (Solute carrier family 34 member 1 = type II sodium/phosphate Na-Pi IIa cotransporter, Taqman prob Mm00441450_m1). Two genes, *Dffa* (DNA fragmentation factor subunit α, Taqman probe Mm01257835_m1) and *Gapdh* (Glyceraldehyde-3-phosphate dehydrogenase, Taqman probe Mm99999915_g1) were used as internal control for more accurate normalization of expression data; cDNAs were amplified using an ABI PRISM_7900 system (Applied Biosystems, Foster City, CA, USA) according to manufacturer’s instructions. Relative Quantification (RQ) of mRNA expression values were calculated by the ΔΔCt method with normalization of each sample to the average change in cycle thresholds of controls using DataAssit v3.01.

**Immunoblotting analyses**

Measures of 50 µg of extracted proteins were separated under denaturing and reducing conditions on a 10% polyacrylamide gel and then transferred to a Hybond C-Extra Nitrocellulose membrane (GE Healthcare, Buckinghamshire, UK). Nonspecific antibody binding was prevented using blocking buffer for 1 h at room temperature with 5% bovine serum albumin in Tris-buffered saline (TBS: 50 mM Tris-HCl, 150 mM NaCl, pH 7.6) for anti-β-ACTIN antibody (C-11; Santa Cruz Biotechnology, Dallas, TX, USA) or with 5% non-fat dry milk in 0.1% Tween 20-TBS (TBST) for anti-PHEX antibody (H-176; Santa Cruz Biotechnology), followed by incubation with the specific primary antibodies overnight at 4 °C. Anti-β-ACTIN and anti-PHEX antibodies were used at a dilution of 1/2000 in 0.05% TBST and 1/1000 in 0.01% TSBT, respectively. After 3 washes of 10 min each, blots were incubated for 1 h at room temperature with the dilution 1/1000 of the corresponding horseradish peroxidase (HRP)-coupled secondary antibody (Dako): Anti-goat IgG (P0449) in 0.05% TBST and anti-Rabbit (P0399) in 2.5% non-fat dry milk in 0.1% TBST for β-ACTIN and PHEX, respectively. After 3 more washes, immunoblots were revealed by enhanced chemiluminescence using BM Chemiluminescence Western blotting substrate (peroxidase (POD)) (Roche Applied Science, Penzberg, Germany) and exposed to a film (Hyperfilm ECL; GE Healthcare, Chicago, IL, USA). All washes were done with 0.05% and 0.1% TBST for β-ACTIN and PHEX immunoblotting, respectively. Films were scanned using LabScan 5 software and the expression was quantified using ImageQuant TL v2005 software.

**Construction of WT-PHEX and PHEX^L222P^ structural models**

Putative models of wildtype PHEX and PHEX^L222P^ were obtained using AlphaFold2, a state-of-the-art machine-learning approach tool for structure prediction [52]. The wildtype structure was directly obtained from the AlphaFold protein structure database (https://alphafold.ebi.ac.uk, accessed on 1 January 2021) [53] using the Unitprot access ID P70669. The PHEX^L222P^ mutant structure was predicted using the full version of AlphaFold software on our computational resources, by mutating L222 into proline. The structure is available in the Supplementary Information. However, it must be stressed that the present structure of PHEX^L222P^ should be considered very carefully since recent studies have pointed out that AlphaFold is unable to accurately predict defective protein folding from single-point mutation [54,55]. Pictures were rendered using the VMD package [56].

## 3. Results

**Generation and phenotype of the PHEX^L222P^ mouse line**

**Figure 1:**
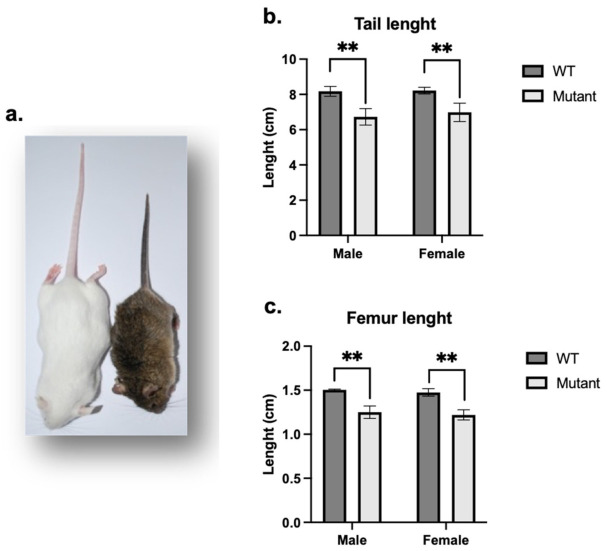
**Whole animal and skeletal characteristics of *Phex^L222P^* versus WT mice.** (**a**) *Phex^L222P^* (right) mice are smaller and have shorter body size than their WT littermates (left). Tail (**b**) and femoral (**c**) lengths of age-matched (3 months), sex-matched mutant (light grey bars) and WT (dark grey bars) mice. Error bars indicate standard deviation. Mutant males are hemizygous (*Phex^L222P^*/Y) and mutant females are heterozygous (*Phex^L222P^*/*Phex*). Mutant and wildtype mice are on a mixed B6/FVB background. One-way ANOVA test was used to compute *p*-values; significance was indicated with asterisks (** *p*-value < 0.01).

*Phex^L222P^* mice were identified in our ENU mutagenesis screen by their smaller body size (Figure 1a), shortened tail and reduced plasma phosphorus levels in males and females. Mutant females were bred with WT FVB males and mutant males were bred with WT FVB females to produce several generations of mice on the FVB background, of which all mutant mice carry the same traits. The *Phex^L222P^* mice showed a smaller body size conserved along the generations. *Phex^L222P^* mouse skeleton measurements of the skeletal preparation showed reduced length of most bones throughout the skeleton, for example, shorter limbs including tibiae and femurs (Figure 1c), and shorter and smaller vertebrae, in particular, the tail vertebrae resulting in a reduced tail length (Figure 1b).

**Figure 2:**
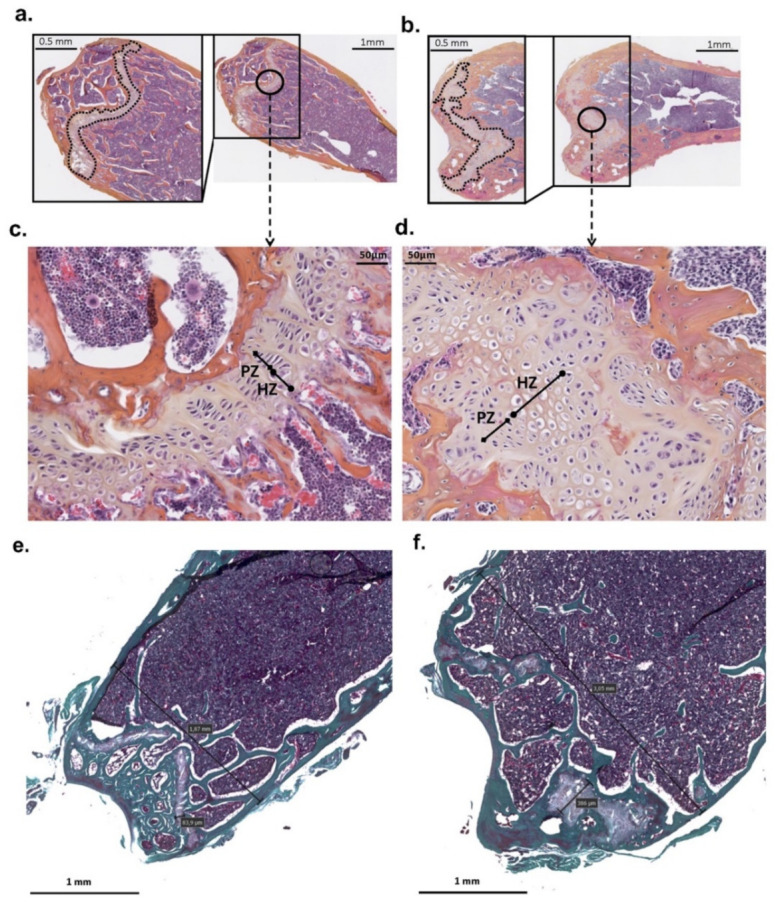
**Distal femur histology of *Phex^L222P^* and WT mice.** The cross-section of the distal femur of mutant male (*Phex^L222P^*/Y) (**b**,**d**,**f**) compared to littermate (*Phex*/Y) control (**a**,**c**,**e**) at 3 months of age. The growth plate is outlined in the magnifications of the boxed area. ***c*** and *d* are a magnification of the circled area in (**a**,**b**), respectively. (**e**,**f**) represent trichrome stains. Scale bars are presented on the figure. PZ: proliferative zone, HZ: hypertrophic zones.

Histological analysis of mutant mice femur showed an increase in their diameter with an enlarged and disorganized epiphyseal growth plate due to increased hypertrophic zones as compared to controls. In addition, osteocyte lacunae are randomly organized compared to those in controls (Figure 2).

**Figure 3:**
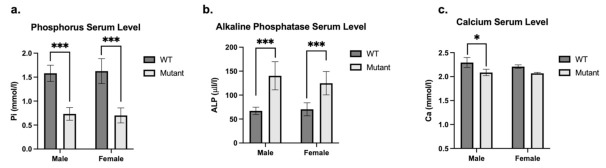
**Plasma biochemical analysis of mutant and WT mice.** Phosphorus (Pi mmol/l) (**a**), Alkaline Phosphatase (ALP U/l) (**b**) and Calcium (Ca mmol/l) (**c**) plasma level of sex-matched mutant (light grey bars) and WT (dark grey bars) mice. Mutant males are hemizygous (*Phex^L222P^*/Y) and mutant females are heterozygous (*Phex^L222P^*/*Phex*). Error bars indicate standard deviation. One-way ANOVA test was used to compute *p*-values, significance was indicated with asterisks (* *p*-value < 0.05; *** *p*-value < 0.001).

Analysis of plasma biochemistry in 8-week-old mice revealed that mutant males and females had significantly reduced plasma phosphate concentrations (*p* < 0.001) (Figure 3a) in association with elevated alkaline phosphatase activity (*p* < 0.001) (Figure 3b) as compared to WT mice. Calcium level is also significantly reduced in mutant males (*p* < 0.001) and females (*p* < 0.5) (Figure 3c) as compared to controls.

**An ENU-Induced, X-Linked dominant mutation affecting the skeleton in Phex^L222P^/Y mice**

**Figure 4:**
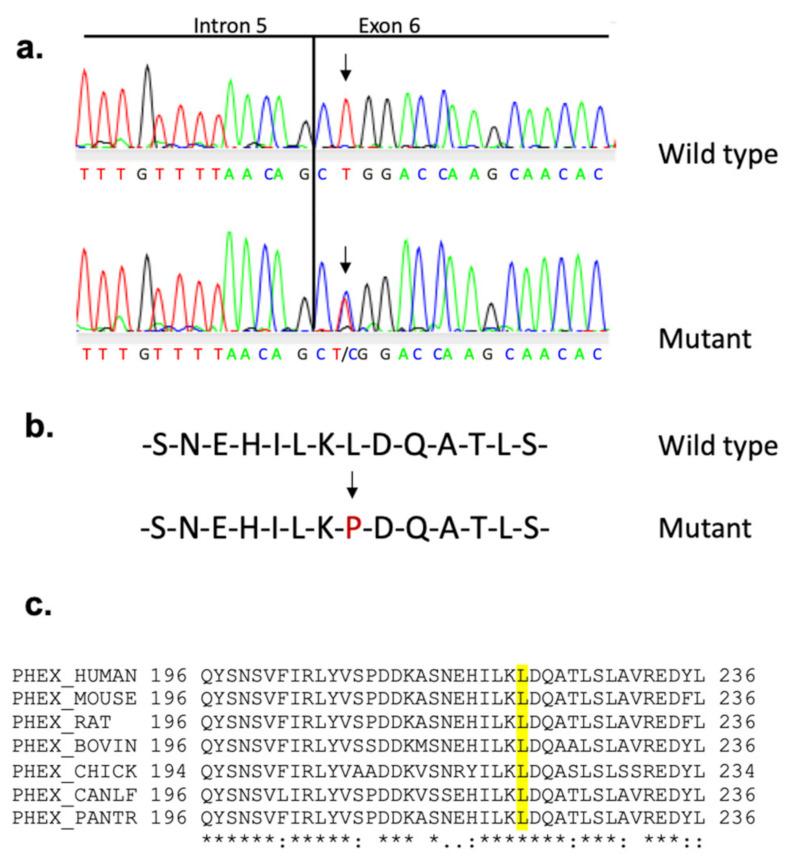
**Sequence analysis spanning *Phex^L222P^* mutation site.** (**a**) Sequence chromatogram of the *Phex* gene at the level of the splicing site intron 5/exon 6 showing the T > C transition (arrows) with the corresponding amino acid sequence in female mouse. (**b**) The resulting PHEX amino acid sequence with the change colored in red. (**c**) Partial protein alignment of PHEX from different species around the mutation position (amino acid 222 in mouse). The leucine residue at position 222, colored in yellow, is highly conserved between the species.

To establish the colony, we mated affected females with WT males and reciprocally affected males with WT females. During this process, it became apparent that although affected females gave rise to approximately equal numbers of affected and unaffected male and female progeny, affected males sired only unaffected male and affected female progeny, suggesting that the skeletal abnormality was inherited in an X-linked dominant trait. To verify the absence of any other mutation, the genetic mapping of affected mice confirmed that the mutation is located on the X-chromosome within a 3 cM interval containing *Phex* gene as candidate. Sequencing of *Phex* cDNA identified a thymine to cytosine replacement (Figure 4a) in the second base pair of exon 6 (c.665T > C), inducing a missense mutation at amino acid 222 (Leu222Pro) (Figure 4b). This mutation generated a replacement of an AluI by an MspI restriction enzyme site at the genomic level, used to confirm that mutant male and female mice were hemizygous (*Phex^L222P^*/Y) and heterozygous (*Phex^L222P^*/*Phex*), respectively. The leucine residue at L222 is highly conserved across different species (Figure 4c). First, to evaluate the potential impact of the mutation, we performed PANTHER-PSEP (position-specific evolutionary preservation), predicting disease-causing genetic variants using position-specific evolutionary preservation (http://pantherdb.org/about.jsp; accessed on 1 January 2019) [57] and PolyPhen analysis, which is used for the prediction of functional effects of human nsSNPs (http://genetics.bwh.harvard.edu/pph2/index.shtml; accessed on 1 January 2019) [58]. These in silico prediction engines show that this mutation is probably damaging with a preservation time of 750 M (>450 million years) and a prediction score of 1.000 (sensitivity: 0.00; specificity: 1.00), respectively (data not shown). The same results are also shown by the mutation analysis by PROVEAN Protein Batch prediction, which provides PROVEAN prediction for all mouse proteins and variants (http://provean.jcvi.org/protein_batch_submit.php?species=mouse; accessed on 1 January 2019).

***Effect of Phex^L222P^ mutation on Phex mRNA/protein, bone and kidney markers expression***

**Figure 5:**
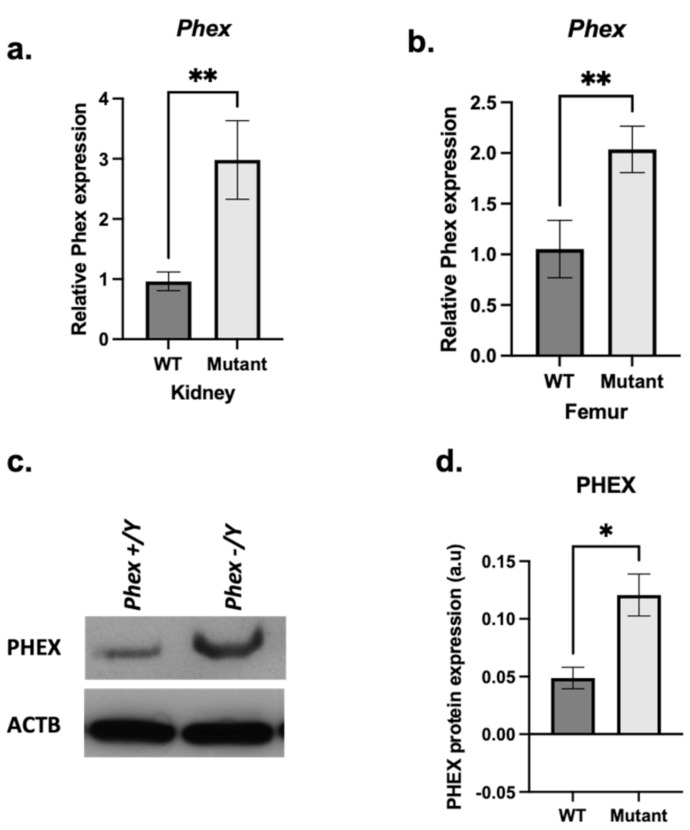
***Phex* mRNA and protein expression.** A comparison between mutant (light grey bars) and WT (dark grey bars) femoral (**a**) and renal (**b**) *Phex* mRNA expression. (**c**) Immunoblot analysis from hemizygous mutant male femurs (*Phex^L222P^*/Y) and WT (*Phex*/Y) littermate using anti-PHEX antibody (*c*-top) and anti-β-ACTIN antibody (*c*-bottom). (**d**) Semi-quantification of PHEX expression after normalisation using anti-β-ACTIN antibody. Samples were taken from male age-matched (3 months old) mutated hemizygous (*Phex^L222P^*/Y) and WT (*Phex*/Y) mouse femurs. Error bars indicate standard deviation. One-way ANOVA test was used to compute *p*-value; significance was indicated with asterisks (* *p*-value <0.05, ** *p*-value < 0.01; n = 3 for each group); ms: marginally significant (*p*-value < 0.07).

The results are limited to hemizygous males to avoid heterozygosity in females. In the femurs of 3 months mutant males, *Phex^L222P^* mRNA was highly expressed (35.5%) compared to that in WT femurs (Figure 5a); this augmentation was also observed at the protein level and confirmed by immunoblotting using anti-PHEX antibodies (Figure 5c,d).

**Figure 6:**
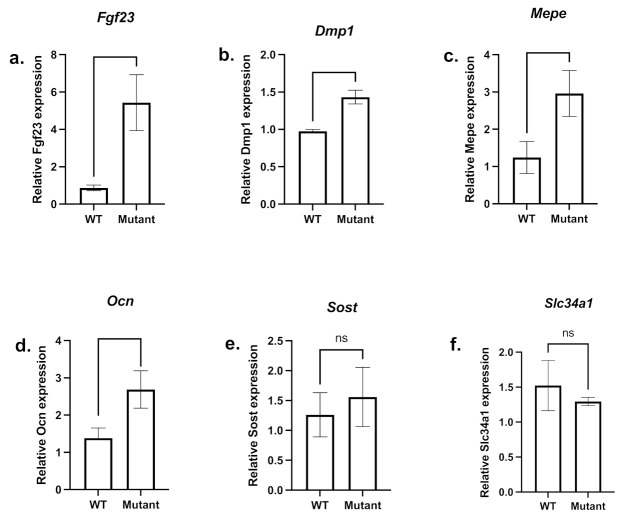
**mRNA expression of femoral bone markers and renal type IIa sodium/phosphate cotransporter.** A comparison between mutant (light grey bars) and WT (dark grey bars) femoral *Fgf23* (**a**), *Ocn* (**d**), and *Sost* (**e**) mRNA expression as well as SIBLINGs *Dmp1* (**b**) and *Mepe* (**c**) and the renal Na-Pi IIa cotransporter (*Slc34a1*) (**f**) mRNA expression level are shown. Samples were taken from male age-matched (3 months old) mutated hemizygous (*Phex^L222P^*/Y) and WT (*Phex*/Y) males. Error bars indicate standard deviation.

However, *Fgf23* mRNA expression was still significantly increased in mutant male femurs (Figure 6a). The analyses of some SIBLING and other matrix protein mRNA expression levels showed a significant increase in *Dmp1* (Figure 6b), *Mepe* (Figure 6c) and *Ocn* (Figure 6d) mRNA expression in mutant male femurs compared to controls. *Sost* expression was not significantly affected (Figure 6e). In addition, mutant male kidneys (Figure 6f) show a slight but not significant decrease in the expression of *Na-Pi IIa* cotransporter.

***Effect of Phex^L222P^ mutation: structural insights***

**Figure 7:**
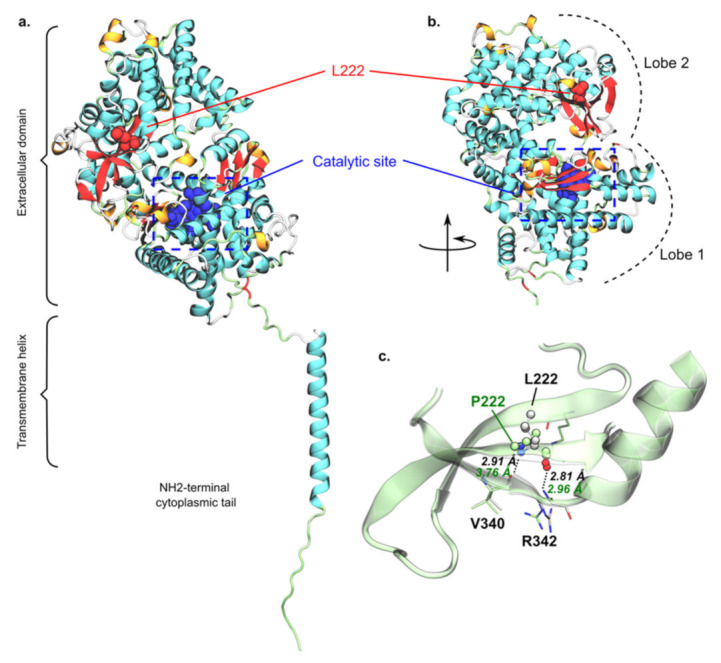
**Predicted structure of PHEX and putative rationalization of L222P mutation.** Overall predicted structure of wildtype PHEX obtained from AlphaFold2 (**a**) as well as the extracellular domain folding into bi-lobe structure *(***b**), in which L222 and the catalytic site are depicted in red and blue, respectively. L/P222 regions of wildtype PHEX and PHEX^L222P^ models were superimposed, (**c**) highlighting key predicted inter-*β*-sheet H-bonds of wildtype PHEX (black) and PHEX^L222P^ (green). Pictures were rendered using the VMD software.

AlphaFold predicted that structures of PHEX and PHEX^L222P^ encompass three main domains, namely a short N-terminal cytoplasmic tail, a transmembrane helix and a large extracellular domain (Figure 7a). Globally, the predicted model exhibited relatively high confidence scores except for the N-terminal domain as well as the loop connecting the transmembrane helix to the extracellular domain. The lower confidence regarding the latter likely pictured higher flexibility of the whole extracellular domain, required for substrate binding prior to PHEX peptidase activity. The extracellular domain adopts a bi-lobe folding in which each subunit is separated by a large cavity to which the catalytic site is exposed (Figure 7b). Structure prediction suggested two lobes, mostly made of *a*-helices. The zinc-center catalytic site was expected to be located in the lobe closer to the membrane. This suggested that substrate-PHEX interactions may trigger the extracellular domain opening at the interface between the two lobes. This in turn may favour substrate binding close to the PHEX catalytic site. The predicted structure of PHEX^L222P^ by AlphaFold does not show significant difference as compared to the wildtype structure, the extracellular domain backbone root-mean-square deviation (RMSD) being only 0.44 Å. This was expected given the known failure of AlphaFold2 to predict defective protein folding from single mutation. Therefore, no clear conclusion can be drawn from the mutant predicted structure.

However, the predicted structure of wildtype PHEX interestingly suggested that L222 is located in the second lobe, at the interface between two pairs of two β-sheets (Figure 7b). This 2 + 2 *β*-sheet arrangement is located on the other side of the inter-lobe cavity as compared to the catalytic site. Moreover, L222 was predicted to interact with V340 and R342 through the interbackbone H-bond network, as usually seen in inter-*b*-sheet arrangement. For instance, interatomic distance between backbone heavy atoms were predicted at 2.91 and 2.96 Å, respectively, for ^L222^O-atom/^R342^N-atom and ^L222^N-atom/^V340^O-atom pairs. In PHEX^L222P^, L222 is mutated into proline, which is known to be a secondary structure breaker [59,60] since the proline backbone forms a ring, which in turn significantly reduces its φ/ψ rotational flexibility. Even though the AlphaFold PHEX^L222P^ structure should be carefully considered, it predicted a significantly larger distance between ^P222^N-atom and ^V340^O-atom (2.91 *versus* 3.76 Å, respectively, for wildtype PHEX and PHEX^L222P^, Figure 7c). Therefore, P222 might locally break the E217-Q224 *β*-sheet, which in turn might disrupt inter-*β*-sheet interactions. We can thus expect a local protein misfolding, which might be responsible for the experimentally observed PHEX^L222P^ loss of function. However, the present hypothesis requires confirmation by further structural investigations such as advanced molecular dynamics simulations.

## 4. Discussion

ENU mutagenesis is a particularly valuable methodology to recover an allelic series of point mutations for any gene enabling a more acute analysis of gene function [61,62,63,64,65,66,67,68]. To date, 441 genes for which mutations result in rare human skeletal disorders are listed by the 2019 International Skeletal Dysplasia Society, from which 260 genes were examined for skeletal phenotypes using mutant mice and 37 phenotypes were successfully identified by ENU mouse mutagenesis [69]. Mutant mice generated by our ENU mutagenized screen present a short body size, hypophosphatemia with consistent hypocalcemia and an increased ALP plasma level. Mutants are found to carry an L222P mutation in the *Phex* gene located on X chromosome. We performed extended outcrossing to remove most of the potential accompanying mutations. In this context, we chose to sequence the entire PHEX gene and not to perform WGS as in many articles using the ENU without WGS [70,71]. The same mutation was detected in one patient for XLH [72]. Normal phosphate, calcium and ALP levels are necessary for bone formation, growth and mineralization. This growth retardation reflects the reduced bone lengths throughout the mutant body, causing growth retardation. The increased thickness of the femur growth plate due to increased hypertrophic chondrocyte zones compared to controls could be caused by the decrease in hypertrophic chondrocyte apoptosis compared to controls [73] and thought to be secondary to rickets [74].

In mouse models, loss of PHEX function or increased FGF-23 production inhibits mineralization mainly, but not only, because of hypophosphatemia due to decreased renal Pi reabsorption, secondary to the decreased abundance of the brush border membrane (BBM)-associated Na-Pi cotransporter proteins [20,75,76,77,78] and the decreased intestinal absorption of phosphate [79]. In addition, PHEX absence or loss of function induces an indirect increase in FGF23 in *Hyp* and *Jrt* [29,32,33] and reduces its degradation [80], thus inhibiting the mineralization indirectly through its effect on serum phosphorus levels via kidney function [81,82] and repressing serum 1,25-dihydroxy-vitamin D. Furthermore, local repression of calcitriol and TNAP could also inhibit mineralization via the upregulation of FGF23 and the loss of degradation of OPN and pASARM by PHEX, alongside the accumulation of pyrophosphate (PPi) [20]. Overexpression of FGF23 has been shown to directly inhibit the mineralization in rat calvaria cell culture [83] and dentin mineralization and dentogenesis in mice [84]. In addition, a FGF23 treatment of primary calvarial osteoblast cultures from WT mice or from the osteoblastic MC3T3-E1 cell line leads to the inhibition of mineralization [85,86]. However, an anti-FGF23 antibody treatment can normalize phosphate and vitamin D metabolism and improve rachitic changes in XLH patients [87,88] and *Hyp* mice [89,90]. In *Phex^L222P^* mice, an increasing *Fgf23* mRNA expression is observed, with a slight but insignificant decrease in the Na-Pi IIa cotransporter mRNA expression level, which could be an indirect cause of hypophosphatemia and so could induce a probable mineralization defect [91]. Furthermore, other pathways could be involved, other than in the renal wasting [20] as the decrease in Na-Pi IIa cotransporter mRNA expression level in our mouse model is not significant. In addition, hypophosphatemia alone is insufficient to explain the bone defect seen in the *Hyp* mouse as the correction of hypophosphatemia failed to correct the mineralization defect observed [35].

Another protein, MEPE, regulates bone mineralization. MEPE null osteoblasts in culture show a dramatic increase in the mineral apposition rate (MAR) [92]; however, the overexpression of MEPE in mice leads to a growth and mineralization defect due to a decrease in bone remodeling. Mice overexpressing MEPE display wider epiphyseal growth plates and expanded primary spongiosa as well as a significant decrease in MAR [93]. MEPE activity is dependent upon ASARM peptide cleavage product as MEPE-ASARM peptides inhibit mineralization both in vivo and in vitro [34,36,94]. The expression of *Mepe* has been shown to be elevated in *Hyp* osteoblasts [95] and *Jrt* bone [33], and it is similarly elevated in *Phex^L222P^* mice.

In *Phex^L222P^* mice, as in *Hyp*, *Dmp1* mRNA expression increases. DMP1 native form inhibits mineralization, but when cleaved into the N-terminal and C-terminal portion by bone morphogenetic protein-1(BMP-1)/Tolloid-like proteinases or when it is dephosphorylated, DMP1 initiates mineralization [96,97]. PHEX is proposed to interact with DMP1 by binding to the DMP1-ASARM motif located at the C-terminal region of DMP1. The PHEX-DMP1 binding initiates a signaling pathway that reduces FGF23 expression and induces mineralization of the growth plate of ossification zone [98,99,100]. However, DMP1 N-terminal fragment localized at the resting, proliferation and pre-hypertrophic zones was demonstrated as biomineralization inhibitor in *Hyp* mice [98,99].

*Ocn* and *Sost* are involved in bone mineralization and their expressions are elevated in *hyp* mice [101,102,103,104] but not in *Phex^L222P^* mice.

In contrast to other mouse models, *Phex^L222P^* expression (mRNA and protein) is increased in this mutant mouse line with a replacement of Leucine (an amino acid with a branched side chain) by a proline (an amino acid with a side chain), which most probably changes the protein conformation, at least locally. This augmentation of PHEX^L222P^ expression induces almost the same phenotypes observed in other XLH mouse models [21,33,44,45,105], which means that PHEX has probably lost its function despite its increased expression. This could be the result of a protein trafficking, endopeptidase activity or the protein conformation problem. The effect analyses of D237G, the closest missense mutation to L222P, show that the protein is secreted (not trapped inside the transfected cells); it also shows that it exhibits 50–60% of wildtype activity using synthetic substrate, in addition to being more resistant to endoproteinase Glu-c than the wildtype protein [18]. This could lead us to think that L222P could probably cause reduced activity of the protein.

## 5. Conclusions

**Figure 8:**
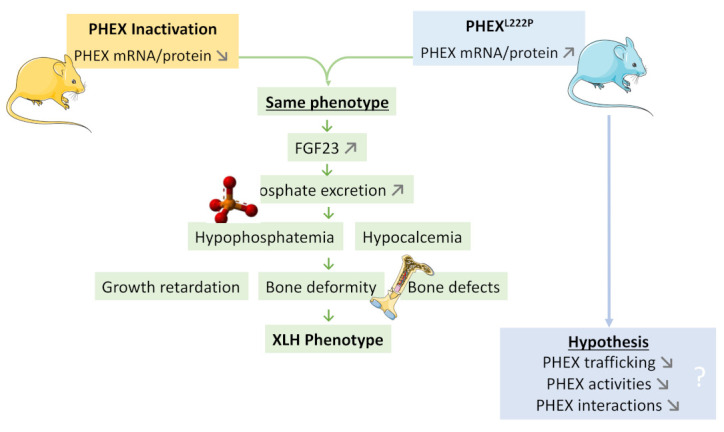
**A model highlighting the Phex^L222P^ mutation in a new ENU mouse model for XLH disease.** *Phex^L222P^* mouse is a new ENU mouse model for XLH disease due to Leu to Pro amino acid modification at position 222 (blue mouse in right). Like PHEX inactivation mouse models (yellow mouse in left), *Phex^L222P^* mouse is characterized by growth retardation, hypophosphatemia, hypocalcemia, reduced body bone length, increased epiphyseal growth plate thickness and femur diameter despite the increase in PHEX expression.

Phex^L222P^ mouse is a new ENU mouse model for XLH disease due to Leu to Pro amino acid modification at position 222. The Phex^L222P^ mouse is characterized by growth retardation, hypophosphatemia, hypocalcemia, reduced body bone length, and increased epiphyseal growth plate thickness and femur diameter, despite the increase in PHEX^L222P^ expression. Actually, Phex^L222P^ mice show an increase in *Fgf23*, *Dmp1*, and *Mepe* and *Slc34a1* (a *Na-Pi IIa* cotransporter) expression similar to those observed in Hyp mice. Femoral osteocalcin and sclerostin and *Na-Pi IIa* renal cotransporter do not show any significant variation in *Phex^L222P^* mice. This model could be a valuable addition to the existing XLH model for further comprehension of the disease occurrence and for testing new therapies (Figure 8).

## Data Availability

Not applicable.
